# Correction: Non-cell-autonomous OTX2 transcription factor regulates anxiety-related behavior in the mouse

**DOI:** 10.1038/s41380-021-01195-x

**Published:** 2021-06-25

**Authors:** Clémentine Vincent, Javier Gilabert-Juan, Rachel Gibel-Russo, Daniel Alvarez-Fischer, Marie-Odile Krebs, Gwenaëlle Le Pen, Alain Prochiantz, Ariel A. Di Nardo

**Affiliations:** 1 Centre for Interdisciplinary Research in Biology (CIRB), CNRS UMR 7241, INSERM U1050, Labex MemoLife, PSL Research University, Collège de France, Paris, France; 2 Institute of Neurogenetics, University of Lübeck, Lübeck, Germany; 3 Laboratoire de Physiopathologie des Maladies Psychiatriques, INSERM U1266, Institut de Psychiatrie et Neurosciences de Paris, Université de Paris, Paris, France; 4 Institut de Psychiatrie, CNRS GDR 3557, Paris, France; 5 Faculté de Médecine, Université de Paris, Pôle Hospitalo-Universitaire Evaluation Prévention et Innovation Thérapeutique, GHU Paris Psychiatrie et Neurosciences site Sainte-Anne, Paris, France; 6 Present Address: Institut NeuroMyoGène, CNRS UMR 5310, INSERM U1217, Université Claude Bernard Lyon 1, Lyon, France; 7 Present Address: Department of Anatomy, Histology and Neuroscience, School of Medicine, Universidad Autónoma de Madrid, Madrid, Spain

**Keywords:** Neuroscience, Biological techniques

Correction to: *Mol Psychiatry* 10.1038/s41380-021-01132-y

The article “Non-cell-autonomous OTX2 transcription factor regulates anxiety-related behavior in the mouse”, written by Clémentine Vincent, Javier Gilabert-Juan, Rachel Gibel-Russo, Daniel Alvarez-Fischer, Marie-Odile Krebs, Gwenaëlle Le Pen, Alain Prochiantz & Ariel A. Di Nardo, was originally published electronically on the publisher’s internet portal on 7 May 2021 without open access. With the author(s)' decision to opt for Open Choice the copyright of the article changed on 10 June 2021 to © The Author(s) 2021 and the article is forthwith distributed under a Creative Commons Attribution 4.0 International License, which permits use, sharing, adaptation, distribution and reproduction in any medium or format, as long as you give appropriate credit to the original author(s) and the source, provide a link to the Creative Commons licence, and indicate if changes were made. The images or other third party material in this article are included in the article’s Creative Commons licence, unless indicated otherwise in a credit line to the material. If material is not included in the article’s Creative Commons licence and your intended use is not permitted by statutory regulation or exceeds the permitted use, you will need to obtain permission directly from the copyright holder. To view a copy of this licence, visit http://creativecommons.org/licenses/by/4.0.

