# Supplementary material for: Non-cell-autonomous OTX2 transcription factor regulates anxiety-related behavior in the mouse
Source: Mol Psychiatry. 2021 May 7;26(11):6469–80. doi: 10.1038/s41380-021-01132-y (PMC8760049; doi:10.1038/s41380-021-01132-y)
Supplement: Supplementary file 1 — Suppl Fig 1 [file 41380_2021_1132_MOESM1_ESM.pdf]

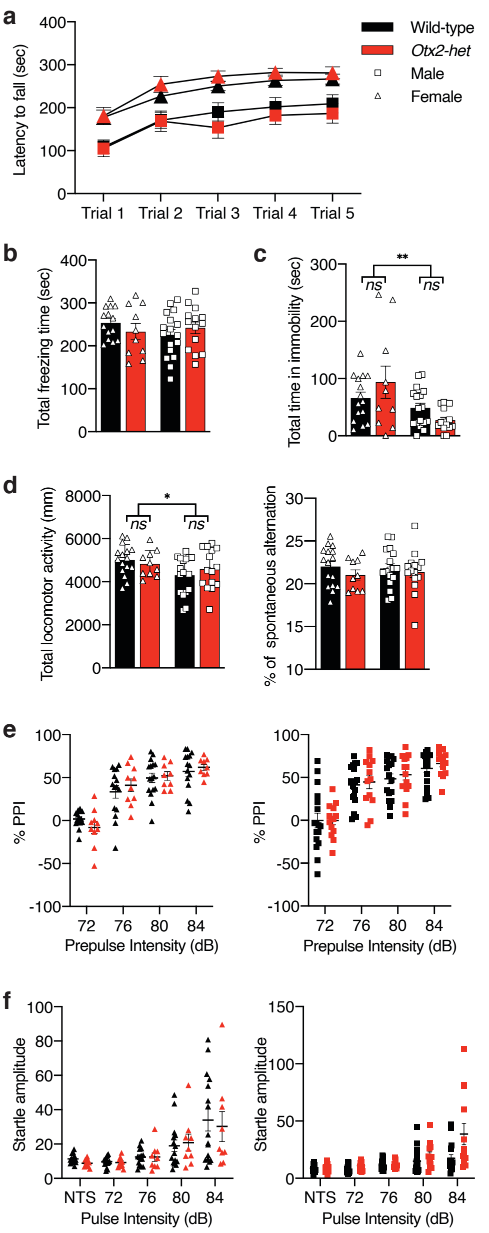

Supplementary Fig. 1 Behavioral analysis of adult male and female *Otx2-het* mice.
a Rotarod test with a progressively accelerating rotating rod from 2 to 40 rpm during 5 min trial (WT females n=16; WT males n=18; *Otx2-het* females n=10; *Otx2-het* males n=14). **b** Forced swim test during a 6 min period in a small pool (WT females n=16; WT males n=17; *Otx2-het* females n=10; *Otx2-het* males n=14). **c** Tail suspension test during a 6 min period (WT females n=16; WT males n=17; *Otx2-het* females n=10; *Otx2-het* males n=15). **d** Y-maze test during a 10 min period (WT females n=16; WT males n=17; *Otx2-het* females n=10; *Otx2-het* males n=15). **e, f** Prepulse inhibition (PPI) test performed in a startle chamber to measure startle response amplitude **f** and %PPI **e** at various pulse intensities (WT females n=16; WT males n=18; *Otx2-het* females n=10; *Otx2-het* males n=14). All values: mean ± SEM; two-way ANOVA, post hoc Tukey test; $*p < 0.05$, $**p < 0.01$.
